# Seasonal Patterns of Soil Bacterial and Fungal Communities Under Practical Organic Fertilizer Regimes in a Tea Plantation

**DOI:** 10.3390/microorganisms14091902

**Published:** 2026-08-27

**Authors:** Yunqi Huang, Mi Hu, Huiting Zhu, Qianwen Sha, Qiaomei Wang, Qiongfen He, Xiujuan Deng, Wenxia Yuan, Yihu Guan, Niuniu Shi, Yapeng Li, Wendi Zhang, Baijuan Wang, Xinghua Wang

**Affiliations:** College of Tea Science, Yunnan Agricultural University, Kunming 650201, China

**Keywords:** tea plantation soil, organic fertilizer regime, bacterial community, fungal community, seasonal variation, soil physicochemical properties, functional prediction

## Abstract

Organic fertilizer and season are established sources of variation in tea plantation soil microbiomes. A repeated-measure field experiment was conducted in Yunnan, China, comprising an unfertilized control (CK), sheep manure (SM), pig manure (PM), rapeseed cake manure (RM), and commercial organic fertilizer (OF). The same 15 mature tea trees were sampled in spring, summer, and autumn, yielding 45 composite soil samples. Soil properties, as well as 16S rRNA gene and ITS amplicon profiles, were evaluated using mixed-effect models, restricted-permutation PERMANOVA, and design-adjusted partial redundancy analysis (pRDA). Fertilization regime, season, and their interaction showed property-specific associations, most clearly for alkali-hydrolyzable nitrogen, available phosphorus, and available potassium. Bacterial alpha diversity was more responsive than fungal alpha diversity. Both factors were significantly associated with bacterial and fungal community structures. Season accounted for most bacterial variation (68.70%), whereas fungal variation was associated more evenly with season (27.33%) and fertilization regime (18.61%); no significant interaction was detected for either group. Nine fungal genera retained significant treatment-associated differences after FDR correction, whereas the bacterial genus-level patterns remained exploratory. After conditioning for fertilization regime and season, measured soil variables collectively explained 6.51% and 13.56% of bacterial and fungal variation, respectively, but no individual variable remained significant after FDR correction. Because fertilizer inputs were not nutrient-standardized, treatment contrasts represent integrated material-and-dose regimes rather than isolated fertilizer-type effects. These context-specific results illustrate the value of repeated seasonal sampling with design-adjusted analyses for evaluating practical fertilization regimes.

## 1. Introduction

Soil quality is fundamental to the long-term productivity, yield stability, and quality formation of tea plants. As a perennial crop, tea plants are subjected to continuous nutrient removal through long-term cultivation and repeated harvesting, making tea plantation management highly dependent on appropriate fertilization practices. Fertilization not only directly affects soil nitrogen, phosphorus, and potassium availability, but also influences soil acidification, organic matter accumulation, and nutrient cycling [1]. Under intensive tea plantation management, long-term inappropriate fertilization, especially excessive chemical fertilizer input, may aggravate soil acidification, reduce nutrient use efficiency, and negatively affect soil ecological functions [2]. In contrast, appropriate organic fertilizer application or organic substitution can increase organic material inputs, improve soil nutrient retention, and provide an important management strategy for maintaining soil health in tea plantations [3].

Soil microorganisms are among the most active biological components of tea plantation soils and play essential roles in organic matter decomposition, nutrient transformation, rhizosphere interactions, and soil health maintenance. Bacteria are generally closely associated with carbon and nitrogen cycling, available nutrient transformation, and changes in the rhizosphere environment, whereas fungi are more involved in the decomposition of complex organic substrates, saprotrophic processes, symbiotic interactions, and potential pathogenic processes. Core bacterial taxa play important roles in organic matter decomposition, nutrient release, and agroecosystem productivity [4]. Therefore, examining both bacterial and fungal communities is necessary for a comprehensive assessment of microbial responses to fertilization management in tea plantations.

Previous studies have compared tea plantation microbial communities under a range of fertilization and organic-management strategies [5]. Integrated organic–chemical fertilization can improve soil properties and alter microbial communities [6]. More recent comparisons of mineral, organic, and combined fertilization regimes have shown that these practices can restructure rhizosphere microbial composition and predicted functions [7]. The combined application of rapeseed cake and green manure also modifies soil nutrient availability and microbial community composition [8]. Similarly, comparisons among leguminous green-manure intercrops have revealed fertilizer-associated differences in soil properties and bacterial diversity [9]. A 2026 field study further showed that microbial inoculants modified the effects of organic fertilizer on soil physicochemical properties, microbial assembly, and tea yield in a cultivar-dependent manner [10]. Together, these findings indicate that microbial responses to organic amendments and fertilizer formulation are already well documented in tea plantations. However, organic fertilizers differ substantially in nutrient composition, carbon-to-nitrogen ratio, mineralization rate, and substrate availability, while practical application rates are often determined by local experience and field operations rather than standardized to equal nitrogen, phosphorus, potassium, or organic carbon inputs. Comparisons among practical organic fertilizer treatments therefore reflect the combined effects of fertilizer source, application rate, nutrient release, and substrate characteristics.

Seasonal dynamics have also been documented in tea plantation soils. A 2025 study of karst tea plantations found that sampling season, together with management practice, affected microbial community structure, functional-gene profiles, extracellular enzyme activities, and ecological stoichiometry [11]. Across spring, summer, and autumn at three tea farms, bacterial composition differed mainly among farms, whereas fungal composition exhibited pronounced seasonal variation [12]. Microbiome composition also varies among ancient tea plantations [13]. Rhizosphere and bulk soils from conventional and ancient tea plantations represent additional distinct microbial niches [14]. These observations show that temporal variation, spatial heterogeneity, and management context are established sources of variation in tea plantation microbiomes. In field fertilization experiments, reliance on a single sampling date may therefore obscure treatment-associated differences against seasonal background variation. Repeated sampling of the same experimental units provides a more appropriate framework for evaluating fertilizer-regime effects while accounting for seasonal dependence within individual trees.

Against this background, the present study focuses on a production-relevant comparison within a specific regional context. An unfertilized control was compared with four locally used organic fertilization regimes, including sheep manure, pig manure, rapeseed cake manure, and a commercial organic fertilizer. The same individual tea trees were repeatedly sampled in spring, summer, and autumn. Mixed-effects models, restricted-permutation PERMANOVA, and design-adjusted partial redundancy analysis (pRDA) were used to account for within-tree dependence and to evaluate fertilization effects in the context of seasonal variation. Accordingly, this study examined how soil-available nutrients, bacterial and fungal community structures, treatment-associated taxa, and predicted functional profiles varied under these practical field fertilization regimes.

Specifically, this study aimed to answer the following questions: (1) How do soil physicochemical properties and bacterial and fungal community structures vary among practical organic fertilizer regimes and across seasons? (2) Do bacterial and fungal communities differ in their responses to fertilization regime and season? (3) To what extent are soil physicochemical properties, collectively and individually, associated with bacterial and fungal community variation after accounting for fertilization regime and season? (4) Are variations in microbial community composition accompanied by corresponding shifts in predicted functional profiles across fertilization regimes and seasons? By addressing these questions, this study provides field-based evidence that may inform context-specific organic fertilizer management, seasonal microbial assessment, and soil health maintenance in tea plantations.

## 2. Materials and Methods

### 2.1. Site Description and Experimental Design

All field experiments were conducted in a tea garden located in Fengqing County, Lincang City, Yunnan Province, China (23°05′ N, 98°40′ E), with an altitude of 1578 m. The experimental region has a mid-subtropical monsoon climate, with a mean annual temperature ranging from 13 °C to 15 °C and an annual precipitation of approximately 1200 mm. The experimental plants were mature individual tea trees of *Camellia sinensis* var. *assamica* that were more than 40 years old.

A total of 15 tea trees growing under comparable topographic and soil conditions were selected for the experiment. A completely randomized design was adopted to assign these plants to five fertilization treatments, including no fertilization control (CK), sheep manure treatment (SM), pig manure treatment (PM), rapeseed cake fertilizer treatment (RM), and commercial special organic fertilizer treatment for tea gardens (OF). Each treatment consisted of three individual tea plants, with each plant serving as an independent experimental unit and one biological replicate.

Fertilizers were applied once on 15 January 2025. The application rates for CK, SM, PM, RM and OF treatments were 0, 15, 20, 20, and 2.5 kg·tree^−1^, respectively. Sheep manure and pig manure were locally sourced in Fengqing County, Yunnan, China and naturally composted to full maturity, the rapeseed cake fertilizer was obtained from Fengqing County Chunju Breeding Farm (Fengqing County, Yunnan, China), and the commercial organic fertilizer formulated for tea plants was obtained from Lincang Enbo Bio-Agriculture Co., Ltd. (Lincang, Yunnan, China). All rates refer to the masses weighed at application and were not converted to dry-matter equivalents. The rates were established before the experiment by the tea plantation production manager according to routine local practice. During application, ditches approximately 20 cm deep and 20 cm wide were dug along the edge of the canopy projection of each experimental tea tree. The assigned fertilizer was evenly distributed in the ditches, which were then covered with soil. Further details are provided in Table S3.

No fertilizer composition was independently measured, and reliable manufacturer- or supplier-guaranteed values for individual N, P_2_O_5_, K_2_O, total nutrients, or organic matter were unavailable. Consequently, the treatments were not equalized for fertilizer mass or N, P, K, or organic carbon inputs, and treatment contrasts should be interpreted as integrated responses to practical per-tree fertilization regimes rather than as isolated effects of fertilizer type or individual nutrients.

### 2.2. Soil Sampling

Soil sampling was performed on the same batch of tested tea plants on 9 April, 1 July, and 28 October 2025, corresponding to the spring, summer, and autumn sampling periods, respectively. For each individual tea plant, five soil samples were collected from the 0–20 cm soil layer at multiple random positions within the canopy projection range. All sampling points were set approximately 10 cm away from the fertilization ditches to avoid interference from fertilization operations, and the five soil samples collected from each tree were thoroughly mixed to form one composite soil sample.

In total, 45 composite soil samples were obtained in this experiment, representing five fertilization treatments, three independent tea plant replicates per treatment, and three sampling seasons. Repeated seasonal observations allowed temporal patterns within individual tea plants to be assessed but did not increase the number of independent experimental units per fertilization treatment. Accordingly, taxon-level differences associated with fertilization should be interpreted with caution, as each treatment comprised three independent tea-plant biological replicates.

After collection, all soil samples were preliminarily processed by removing stones, plant roots, and visible residual plant debris using a 2 mm sieve, and subsequently divided into two parts. One subsample was immediately transferred to sterile centrifuge tubes and stored at −80 °C for subsequent microbial DNA extraction and amplicon sequencing. The other subsample was naturally air-dried at room temperature and sieved through a 2 mm mesh for the determination of soil physicochemical properties. Given that repeated sampling was conducted on the same individual tea plants across spring, summer and autumn seasons, individual plant IDs were included in the statistical model to correct for the repeated measurement structure in subsequent data analysis.

### 2.3. Soil Physicochemical Analysis

Soil physicochemical properties were determined by Yunnan Quanyin Biotechnology Co., Ltd. (Kunming, Yunnan, China). Air-dried soil samples were used to measure pH, soil organic matter (SOM), total nitrogen (TN), total phosphorus (TP), total potassium (TK), alkali-hydrolyzable nitrogen (AN), available phosphorus (AP), and available potassium (AK).

The measurements were performed according to the corresponding standard methods: pH was determined according to NY/T 1377-2007 [15], SOM according to NY/T 1121.6-2006 [16], TN according to NY/T 1121.24-2012 [17], TP according to LY/T 1232-2015 [18], TK according to LY/T 1234-2015 [19], AN according to DB51/T 1875-2014 [20], AP according to NY/T 1121.7-2014 [21], and AK according to NY/T 889-2004 [22]. SOM, TN, TP, and TK were expressed as g kg^−1^, whereas AN, AP, and AK were expressed as mg kg^−1^.

### 2.4. Soil Microbial DNA Extraction, Amplification, and Sequencing

Soil samples for microbial community analysis were stored at −80 °C and sent to a professional sequencing service provider for soil microbial DNA extraction, PCR amplification, library construction, and high-throughput sequencing. Referring to a similar study on tea plantation soil microbiota, microbial genomic DNA was extracted using the E.Z.N.A.^®^ Soil DNA Kit (Omega Bio-tek, Norcross, GA, USA). DNA quality was assessed by agarose gel electrophoresis, and DNA concentration and purity were determined using a NanoDrop 2000 spectrophotometer (Thermo Fisher Scientific Inc., Waltham, MA, USA). Qualified DNA samples were used for subsequent bacterial 16S rRNA gene and fungal ITS amplicon sequencing.

For bacterial community analysis, the V4 region of the 16S rRNA gene was amplified using primers 515F/806R:5′-GTGCCAGCMGCCGCGGTAA-3′ and 5′-GGACTACHVGGGTWTCTAAT-3′. For fungal community analysis, the ITS1 region was amplified using primers 1737F/2043R: 5′-GGAAGTAAAAGTCGTAACAAGG-3′ and 5′-GCTGCGTTCTTCATCGATGC-3′. PCR amplification was performed according to the standard protocol of the sequencing service provider. Negative controls were included in each PCR batch by replacing template DNA with ddH_2_O. Only PCR products without visible amplification in the negative controls were used for subsequent library preparation.

After quality assessment, PCR products were pooled and purified. Amplicon libraries were constructed through end repair, A-tailing, adapter ligation, and purification. Library quality was evaluated by AATI analysis to assess fragment integrity and insert size, and by qPCR to determine the effective library concentration. Qualified libraries were pooled according to their effective concentrations and the target sequencing depth. The bacterial 16S rRNA gene and fungal ITS1 amplicon libraries were sequenced separately on an Illumina NovaSeq 6000 platform (Illumina, San Diego, CA, USA) using a 2 × 250-bp paired-end configuration. The same 45 soil samples were analyzed for both marker genes. Following quality filtering and chimera removal, the mean sequencing depths were 106,250 effective sequences per sample for the bacterial 16S rRNA gene dataset (range, 58,195–178,985; *n* = 45) and 107,634 effective sequences per sample for the fungal ITS dataset (range, 44,541–215,928; *n* = 45).

### 2.5. Data Processing and Statistical Analysis

Raw paired-end reads were demultiplexed by barcode, trimmed of barcodes and primer sequences, merged, quality-filtered, and chimera-filtered to obtain effective sequences for downstream analysis. Processing was performed mainly in QIIME 2 (version 2022.2.1) [23], with amplicon sequence variants (ASVs) inferred using the q2-dada2 plugin (version 2022.2.0) [24]. Representative ASVs were taxonomically classified using QIIME 2’s classify-sklearn algorithm and a pre-trained Naive Bayes classifier against SILVA 138.1 and UNITE v9.0 for bacterial 16S rRNA gene and fungal ITS sequences, respectively [25,26]. Because the 515F/806R primer pair can also amplify archaeal sequences, only ASVs assigned to Bacteria were retained from the 16S dataset, consistent with the study’s focus on bacterial communities; fungal analyses used the ITS ASV feature table.

Alpha diversity was evaluated using the Shannon and Chao1 indices, and sequencing coverage using Good’s coverage. Soil physicochemical properties and alpha diversity indices were analyzed using linear mixed-effects models with fertilizer regime, season, and their interaction as fixed effects and TreeID as a random intercept [27]. For AN, AP, and AK, residual diagnostics guided scale transformation and heterogeneous variance structures. Treatment differences within each season were evaluated using Tukey-adjusted pairwise comparisons (*p* < 0.05).

Beta diversity was assessed using principal coordinate analysis (PCoA) based on Bray–Curtis dissimilarities calculated from Hellinger-transformed genus-level relative abundance matrices [28]. PERMANOVA was used to test the effects of fertilization regime, season, and their interaction [29] using term-specific restricted permutation consistent with the repeated-measures design [30]. The fertilization-regime effect was tested by permuting the intact three-season sample sets of individual tea trees among treatments, whereas season and interaction effects were tested by restricting permutations within TreeID. All tests used 9999 permutations. Homogeneity of multivariate dispersion was evaluated using betadisper, with distances calculated to the spatial median, to determine whether PERMANOVA results were influenced by differences in within-group dispersion [31].

An exploratory screen for treatment-associated genera was conducted using genus-level relative abundance matrices. Kruskal–Wallis tests were first used to compare genus-level relative abundances among organic fertilizer regimes, and Benjamini–Hochberg correction was applied separately to bacterial and fungal genera to calculate FDR-adjusted *p*-values [32]. Genera with FDR-adjusted *p*-values < 0.05 were considered to show statistically significant treatment-associated differences. Genera with Kruskal–Wallis *p* < 0.05 that showed a clear treatment-enrichment pattern and a consistent association direction in at least two seasons, but that did not remain significant after FDR correction (q ≥ 0.05), were retained only as exploratory treatment-associated observations and were not interpreted as statistically significant differential genera.

Partial redundancy analysis (pRDA) related standardized soil physicochemical variables to Hellinger-transformed bacterial or fungal genus-level relative-abundance matrices while accounting for the experimental design. Variables with variance inflation factors (VIFs) > 10 were iteratively removed; thus, TN was excluded, leaving pH, SOM, TP, TK, AN, AP, and AK. Fertilization regime and season were used as conditioning variables, and the overall models and marginal effects of individual soil variables were tested using 9999 permutations restricted within TreeID. Marginal *p*-values were adjusted separately for bacterial and fungal communities using the Benjamini–Hochberg FDR procedure. Sensitivity analysis conditioning on TreeID and season assessed robustness. Potential bacterial functional profiles were predicted with PICRUSt2 (version 2.6.2) from the bacteria-only 16S rRNA gene feature table after removal of archaeal ASVs [33], whereas fungal trophic modes were annotated with FUNGuild (version 1.1) based on ITS taxonomic results [34]. These predictions were used only to describe relative trends and not as direct measurements of functional-gene abundance or metabolic activity.

Except for basic sequence processing by the sequencing service provider, statistical analyses and data visualization were conducted mainly in R version 4.5.2 [35] within RStudio 2026.07.0 + 139 [36]. Linear mixed-effects models were fitted using the nlme (version 3.1-168) [37], lme4 version 2.0-1 [27] and lmerTest (version 3.2-1) [38] packages. The heteroscedastic mixed-effects models for AN, AP, and AK were fitted using the nlme package. Restricted-permutation PERMANOVA, betadisper analysis, and pRDA were conducted using the vegan(version 2.7-2) [39] and permute (version 0.9-8) [40] packages. Figures were generated using ggplot2 (version 4.0.3) [41], pheatmap (version 1.0.13) [42], and related R packages. PERMANOVA results are reported as degrees of freedom (df), sums of squares (SS), R^2^, pseudo-F-values, and *p*-values.

## 3. Results

### 3.1. Effects of Organic Fertilizer Application Regime and Seasonal Variation on Soil Physicochemical Properties

#### 3.1.1. Overall Soil Physicochemical Characteristics

**Figure 1:**
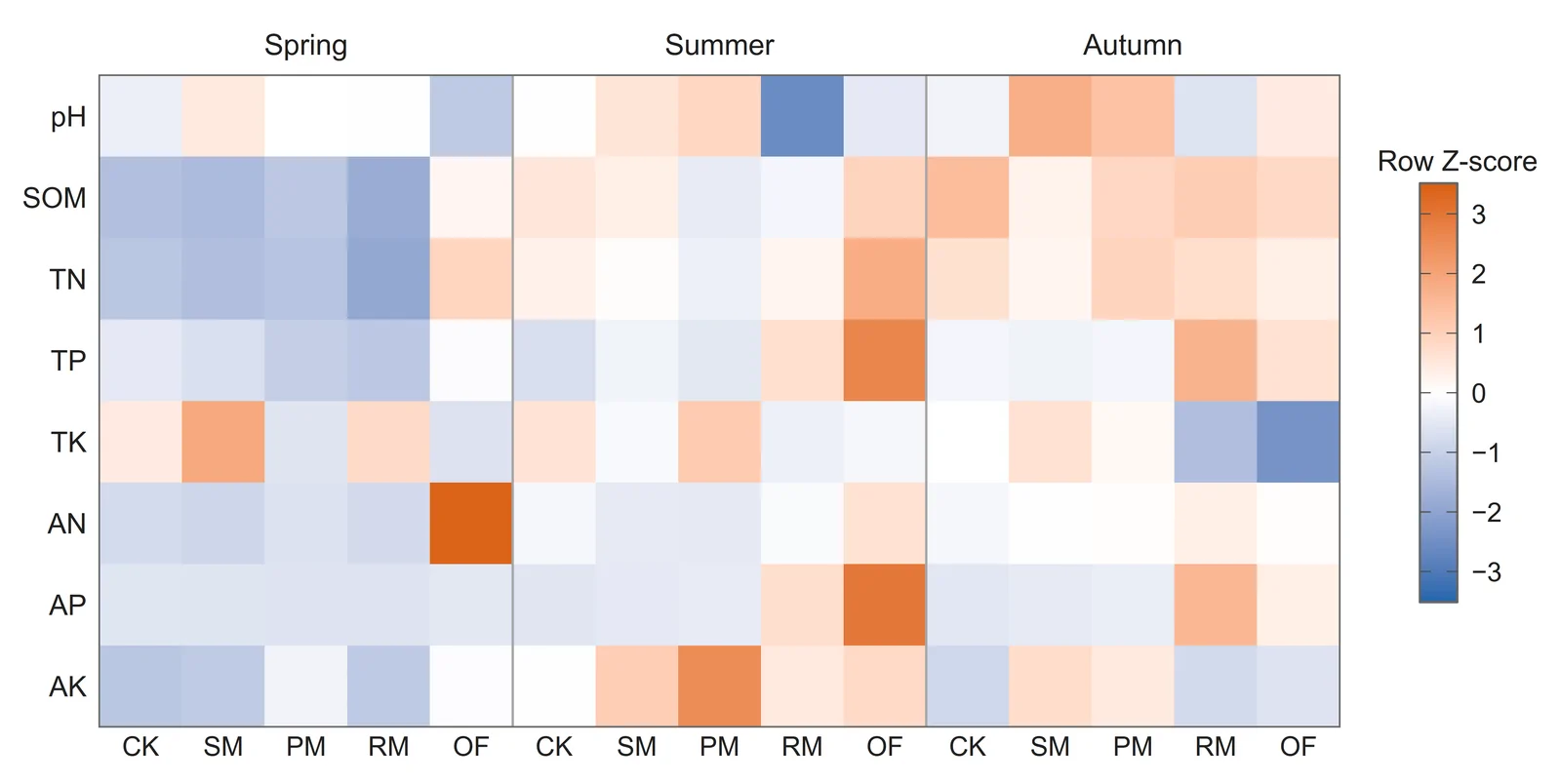
Heatmap of soil physicochemical properties under different organic fertilizer regimes across seasons. Rows represent eight soil physicochemical properties, and columns represent 15 season × fertilization regime combinations. Values are shown as row-wise Z-scores, with the color scale centered at zero; warm and cool colors indicate values above and below the row mean, respectively. CK, control; SM, sheep manure; PM, pig manure; RM, rapeseed cake manure; OF, commercial organic fertilizer.

**Figure 2:**
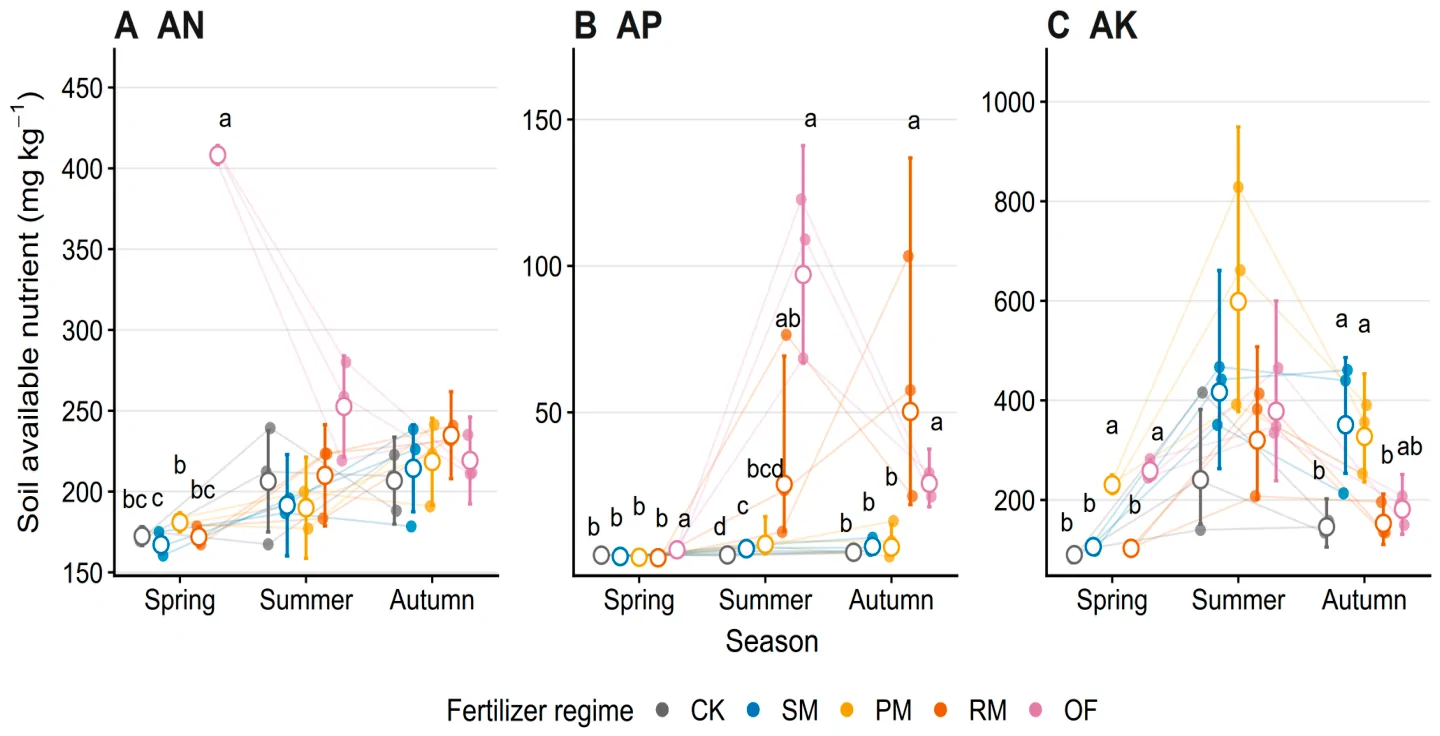
Effects of organic fertilizer regimes and season on key available nutrient indicators in tea plantation soil. (**A**) Alkali-hydrolyzable nitrogen (AN); (**B**) available phosphorus (AP); (**C**) available potassium (AK). Filled points represent individual biological replicates (*n* = 3 per treatment × season), thin lines connect repeated measurements from the same tree, and open points with error bars show estimated marginal means (EMMs) with 95% confidence intervals (CIs). Different lowercase letters indicate significant differences among fertilizer regimes within each season based on Tukey-adjusted pairwise comparisons (*p* < 0.05); letters are omitted when no pairwise differences are significant. CK, control; SM, sheep manure; PM, pig manure; RM, rapeseed cake manure; OF, commercial organic fertilizer.

Across the 15 season × fertilization-regime combinations, mean soil pH ranged from 4.19 to 5.13, indicating consistently acidic soil conditions. Mean SOM, TN, TP, and TK ranged from 37.05 to 55.88, 2.06 to 3.36, 0.54 to 1.27, and 18.15 to 20.02 g kg^−1^, respectively. Mean AN, AP, and AK ranged from 167.13 to 408.28, 0.37 to 100.03, and 90.30 to 627.16 mg kg^−1^, respectively (Figure 1 and Figure 2). Overall, available nutrient pools, particularly AP and AK, varied markedly among season × fertilization-regime combinations, whereas TK remained within a comparatively narrow range.

#### 3.1.2. Effects of Fertilization Regime, Season, and Their Interaction on Soil Physicochemical Properties

The linear mixed-effects models revealed differential responses of soil physicochemical properties to organic fertilization regime and seasonal variation (Table 1). Soil pH, TP, AP, and AK were significantly affected by fertilization regime, season, and their interaction (*p* < 0.05). AN was significantly influenced by fertilization regime and the fertilization regime × season interaction (both *p* < 0.001), whereas the main effect of season was not significant (*p* > 0.05). SOM and TN showed significant main effects of fertilization regime and season, but their interaction was not significant. In contrast, TK showed no significant response to any of the fixed effects included in the model. Among all measured soil properties, AN exhibited the strongest fertilization-regime effect (F = 117.568, *p* < 0.001), together with a significant fertilization regime × season interaction (F = 20.453, *p* < 0.001), indicating that differences in AN among fertilization treatments were strongly dependent on the sampling season.

The heatmap further illustrated variation in soil physicochemical properties across the season × fertilization regime combinations (Figure 1). In spring, soils under the OF treatment exhibited a markedly higher relative AN level. In summer, the OF treatment was characterized by relatively high TP and AP levels, whereas the PM treatment showed the highest relative AK level. In autumn, soil pH was relatively high under the SM and PM treatments, while the RM treatment exhibited relatively high TP and AP levels. SOM and TN contents were also generally higher than those observed in spring across several treatment groups. Because each soil property was independently standardized using row-wise Z-scores, the heatmap represents overall patterns and relative differences among groups rather than differences in absolute values. Therefore, statistical significance should be interpreted in conjunction with the results of the mixed-effects models and the corresponding multiple-comparison analyses.

Further comparisons of AN, AP, and AK revealed that treatment differences varied among seasons and nutrient indices (Figure 2). For AN, the OF treatment showed a significantly higher value than all other treatments in spring, followed by PM. CK and RM exhibited intermediate values, whereas SM had the lowest value. Although AN varied among treatments in summer and autumn, no significant differences among fertilization regimes were detected within either season. For AP, the OF treatment showed a significantly higher value than all other treatments in spring. In summer, OF was significantly higher than CK, SM, and PM but did not differ significantly from RM. RM was significantly higher than CK and SM, whereas PM did not differ significantly from CK, SM, or RM. In autumn, AP was significantly higher under both RM and OF than under CK, SM, and PM, with no significant difference between RM and OF. For AK, the PM and OF treatments exhibited significantly higher values than CK, SM, and RM in spring. No significant differences among fertilizer regimes were detected in summer. In autumn, SM and PM showed significantly higher AK values than CK and RM, whereas OF showed an intermediate response and did not differ significantly from either group.

Taken together, under the practical field application rates used for individual tea trees, soil physicochemical properties responded differently to organic fertilization regime and seasonal variation, with the magnitude and pattern of these responses differing among soil indices. Soil pH, SOM, TN, TP, and several available nutrient indices were influenced by fertilization regime, seasonal conditions, or both. In particular, AN, AP, and AK showed clear season-dependent responses to fertilizer regime, providing important environmental context for the subsequent interpretation of changes in soil microbial communities.

### 3.2. Sequencing Depth and Coverage

Before evaluating microbial diversity and community composition, we assessed the sequencing depth and coverage of the bacterial 16S rRNA gene and fungal ITS datasets. After quality filtering, each sample retained 58,195 to 178,985 valid 16S rRNA gene sequences and 44,541 to 215,928 valid ITS sequences (Tables S1 and S2). Good’s coverage for the bacterial and fungal ASV datasets ranged from 0.993 to 1.000, and the rarefaction curves approached asymptotes, indicating that the sequencing depth was sufficient to characterize microbial community diversity (Figure S1). By contrast, the ASV accumulation curves did not reach full saturation, implying that additional sampling might recover more low-abundance or rare ASVs (Figure S2). Among the ASVs in the filtered 16S table, 809 were assigned to Archaea, representing 441,082 reads (10.17% of the reads in the table; sample range, 0.62–34.69%). Restricting the dataset to ASVs assigned to Bacteria yielded a bacteria-only table containing 39,248 ASVs and 3,894,137 reads for all downstream bacterial analyses.

### 3.3. Structure and Composition of Bacterial and Fungal Communities Across Organic Fertilizer Regimes and Seasons

**Figure 3:**
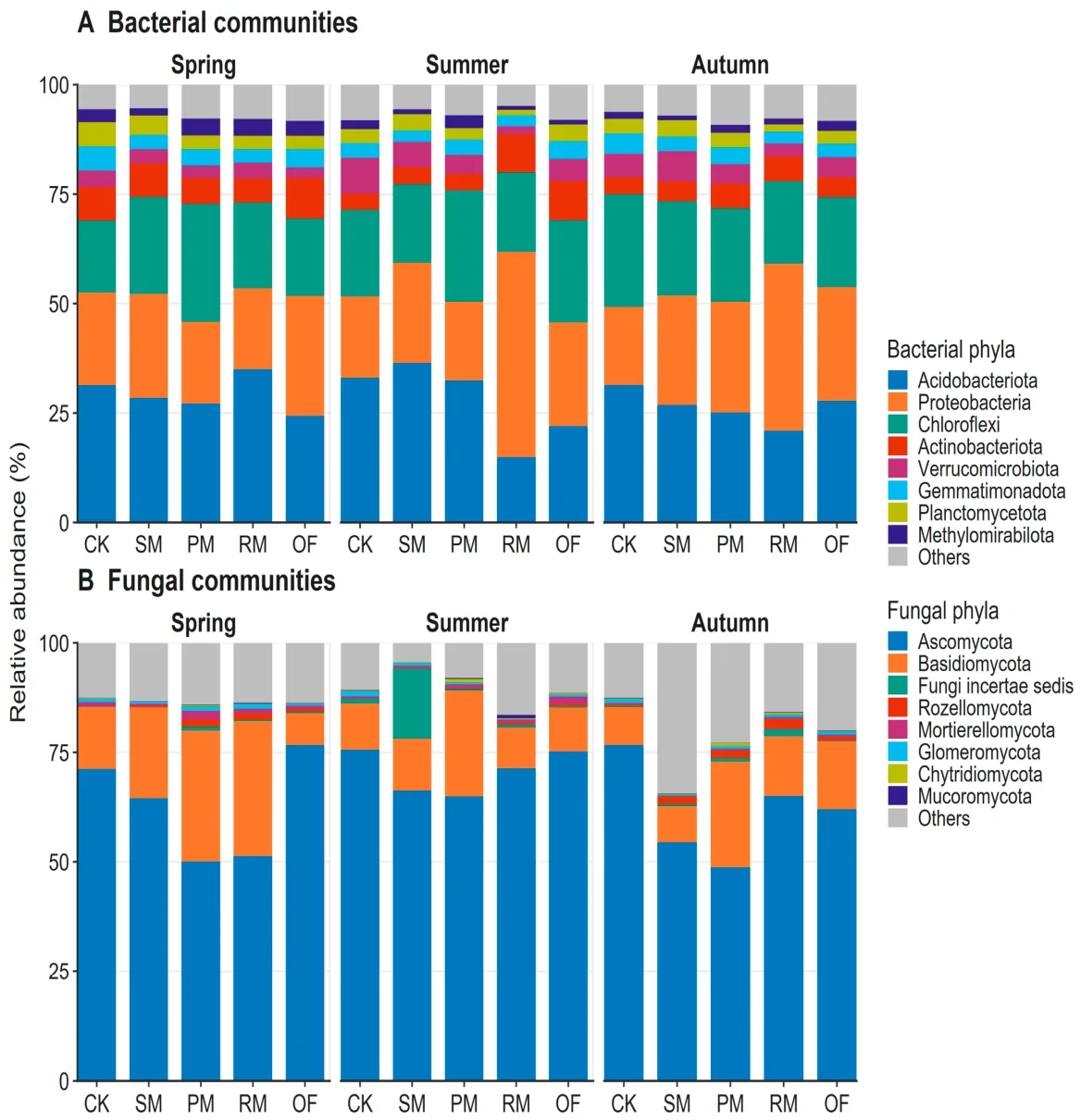
Phylum-level composition of bacterial (**A**) and fungal (**B**) communities under five fertilizer regimes across three seasons. Each 100% stacked bar represents the mean relative abundance of three biological replicates. The eight most abundant phyla in each microbial group are shown individually, and the remaining taxa are pooled as “Others”. CK, control; SM, sheep manure; PM, pig manure; RM, rapeseed cake manure; OF, commercial organic fertilizer.

To characterize the overall taxonomic structure of the soil microbial communities, we compared the phylum-level relative abundance profiles of bacterial and fungal communities across the five fertilization regimes and three seasons (Figure 3). Archaeal sequences were removed from the 16S rRNA gene dataset before bacterial community profiling, whereas fungal community composition was characterized using the ITS dataset.

After the removal of archaeal sequences, the bacterial communities were dominated by Acidobacteriota, Proteobacteria, and Chloroflexi, with mean relative abundances of 27.81%, 24.82%, and 20.98%, respectively. Together, these three phyla accounted for 68.91–79.98% of the bacterial communities across seasons and fertilization treatments (Figure 3A). In contrast, Actinobacteriota, Verrucomicrobiota, Gemmatimonadota, Planctomycetota, and Methylomirabilota consistently occurred at lower relative abundances. These results indicate that the overall phylum-level framework of the bacterial communities remained relatively stable across fertilization regimes and seasons.

Nevertheless, the relative contributions of the three dominant bacterial phyla varied among season × fertilization regime combinations. In spring, Acidobacteriota was the most abundant phylum under CK, SM, PM, and RM, whereas Proteobacteria showed a slightly higher relative abundance than Acidobacteriota under OF. Chloroflexi was comparatively more abundant under PM. In summer, Acidobacteriota remained dominant under CK, SM, and PM, whereas Proteobacteria predominated under RM. In autumn, Acidobacteriota showed the highest relative abundance under CK, SM, and OF, while Proteobacteria was dominant under PM and RM. Thus, the observed variation in bacterial composition mainly reflected redistribution in the relative abundances of specific dominant phyla rather than replacement of the overall dominant phylum assemblage.

The fungal communities were dominated by Ascomycota and Basidiomycota, with mean relative abundances of 64.91% and 15.98%, respectively, whereas the other fungal phyla occurred at substantially lower relative abundances across most season × fertilization regime combinations (Figure 3B). Ascomycota remained dominant in spring, summer, and autumn, with seasonal mean relative abundances of 62.73%, 70.65%, and 61.36%, respectively. The corresponding seasonal mean relative abundances of Basidiomycota were 20.62%, 13.21%, and 14.11%.

Treatment-associated variation was particularly apparent in several fungal compositional profiles. In spring, Basidiomycota accounted for relatively high proportions under PM and RM. The group annotated as Fungi incertae sedis was most prominent under SM in summer. In autumn, the proportion assigned to “Others” reached a seasonal mean of 20.88% and was particularly high under SM and PM. These profiles therefore indicated greater descriptive variation in fungal community composition among fertilization treatments.

Overall, the bacterial communities maintained a relatively stable phylum-level framework dominated by Acidobacteriota, Proteobacteria, and Chloroflexi across seasons and fertilization regimes, although the relative contributions of these phyla varied. Fungal communities were consistently dominated by Ascomycota and Basidiomycota, but showed greater variation in the proportional distribution of both dominant and less abundant taxa. Thus, bacterial and fungal communities exhibited distinct phylum-level compositional patterns, without an overall replacement of their dominant phyla.

### 3.4. Responses of Bacterial and Fungal α-Diversity and β-Diversity to Organic Fertilizer Application Regime and Seasonal Variation

#### 3.4.1. Effects of Organic Fertilizer Application Regime and Seasonal Variation on Soil Bacterial and Fungal α-Diversity

**Figure 4:**
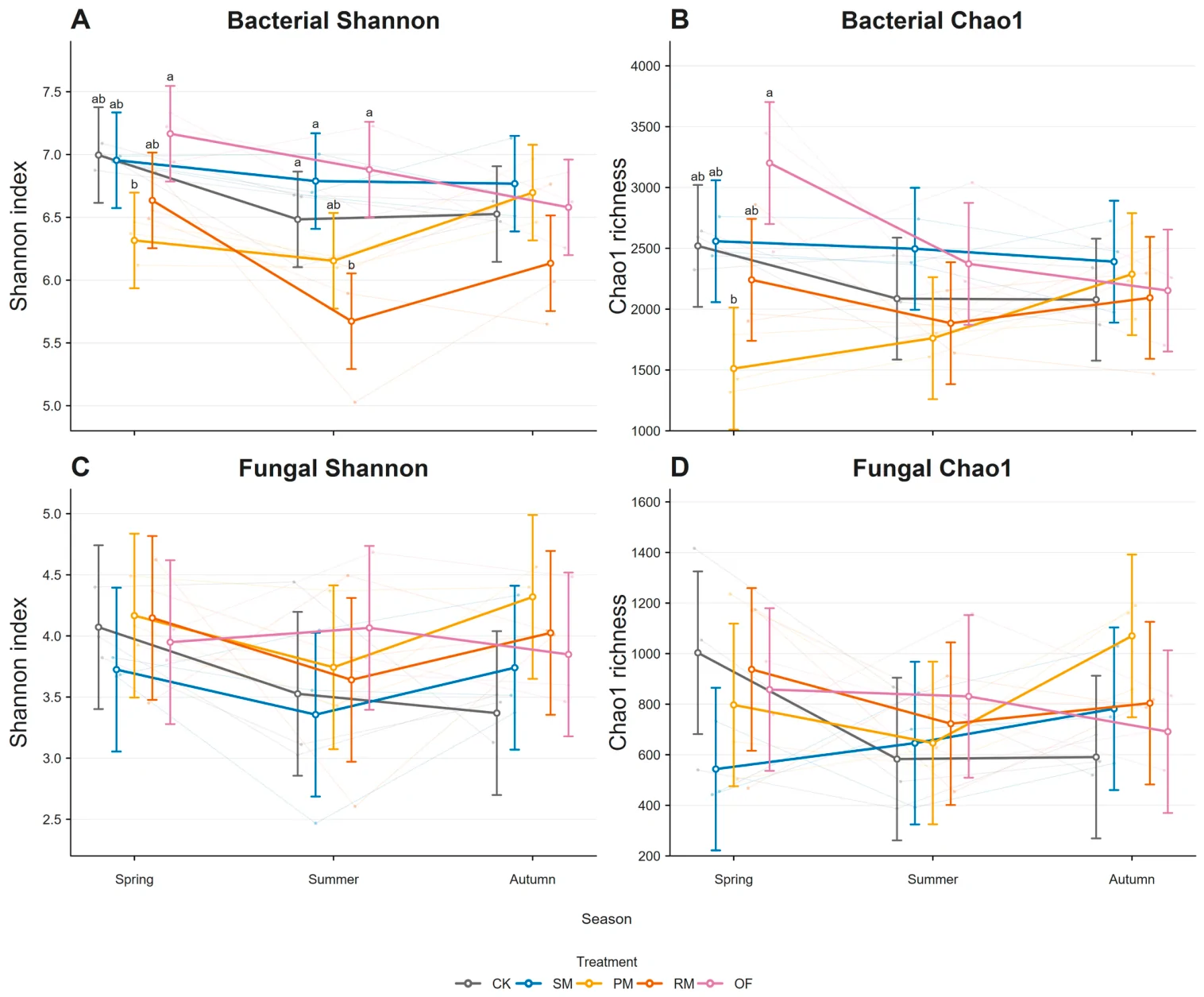
Effects of organic fertilizer regime and seasonal variation on the α-diversity of soil bacterial and fungal communities. (**A**) Bacterial Shannon diversity; (**B**) bacterial Chao1 richness; (**C**) fungal Shannon diversity; and (**D**) fungal Chao1 richness. Open points with error bars show estimated marginal means (EMMs) with 95% confidence intervals (CIs), colored lines connect treatment-level EMMs across seasons, and thin translucent lines connect repeated measurements from the same tree. Different lowercase letters indicate significant differences among fertilizer regimes within the same season (Tukey-adjusted *p* < 0.05). Letters are omitted when no pairwise differences are significant. CK, control; SM, sheep manure; PM, pig manure; RM, rapeseed cake manure; OF, commercial organic fertilizer.

To determine the effects of organic fertilization regime and seasonal variation on soil microbial α-diversity in the tea plantation, the Shannon diversity index and Chao1 richness estimator were calculated from the bacterial 16S ASV table after the removal of archaeal ASVs and from the fungal ITS ASV table. Linear mixed-effects models were subsequently used to evaluate the effects of fertilization regime, season, and their interaction. Fertilization regime and season exerted differential effects on bacterial and fungal α-diversity (Table 2; Figure 4). Bacterial α-diversity indices showed relatively pronounced responses to both fertilization regime and seasonal variation, whereas fungal α-diversity varied only slightly among fertilization treatments and seasons.

Linear mixed-effects modelling showed that bacterial Shannon diversity was significantly affected by organic fertilizer regime (F = 10.537, *p* = 0.0013) and season (F = 7.470, *p* = 0.0038), whereas their interaction was not significant (F = 2.173, *p* = 0.0762) (Table 2). Bacterial Chao1 richness differed significantly among organic fertilizer regimes (F = 10.293, *p* = 0.0014), but not among seasons (F = 1.724, *p* = 0.2038), and was not affected by the organic fertilizer regime × season interaction (F = 1.806, *p* = 0.1354). Thus, fertilizer regime affected both bacterial diversity and richness, whereas the seasonal effect was confined to Shannon diversity.

As shown in Figure 4A,B, significant differences in bacterial α-diversity among fertilizer regimes were observed primarily in spring and summer. In spring, OF showed significantly higher Shannon diversity and Chao1 richness than PM, whereas CK, SM, and RM did not differ significantly from either treatment. In summer, Shannon diversity was lower under RM than under CK, SM, and OF, whereas PM showed an intermediate value and did not differ significantly from the other treatments; Chao1 richness did not differ significantly among fertilizer regimes. In autumn, neither bacterial metric differed among fertilizer regimes. However, the fertilizer regime × season interaction was not significant, indicating that the magnitude of the fertilizer effect did not differ statistically among seasons.

Neither fungal Shannon diversity nor Chao1 richness was significantly affected by fertilizer regime, season, or their interaction (all *p* > 0.05; Table 2). No significant within-season differences among fertilizer regimes were detected for either fungal index. Figure 4C,D therefore showed no consistent differentiation in fungal α-diversity across fertilizer regimes or seasons.

Overall, bacterial α-diversity was more responsive than fungal α-diversity. After archaeal ASVs were removed, bacterial Shannon diversity responded significantly to both fertilizer regime and season, whereas bacterial Chao1 richness responded only to fertilizer regime. In contrast, neither fungal metric exhibited a significant model-level response.

#### 3.4.2. Effects of Organic Fertilizer Application Regime and Seasonal Variation on Soil Bacterial and Fungal β-Diversity

**Figure 5:**
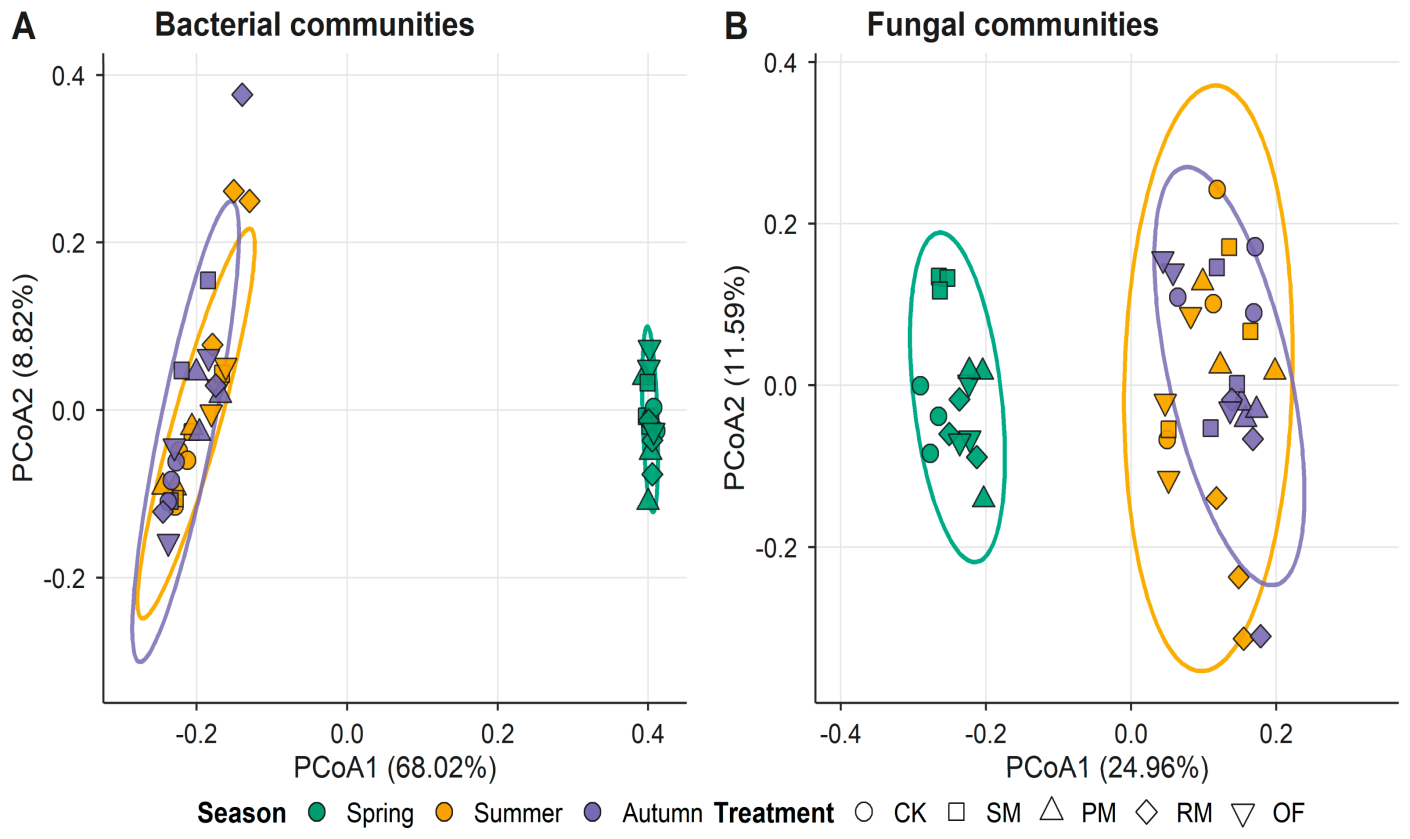
Principal coordinate analysis (PCoA) of soil bacterial and fungal communities across organic fertilizer regimes and seasons. (**A**) Bacterial communities based on the 16S genus-level matrix after removal of archaeal taxa and renormalization; (**B**) fungal communities based on the ITS genus-level matrix. PCoA was performed using Bray–Curtis dissimilarities calculated from Hellinger-transformed genus-level relative abundance matrices. For bacteria, PCoA1 and PCoA2 explained 68.02% and 8.82% of the variation, respectively; for fungi, the corresponding values were 24.96% and 11.59%. Colors denote seasons, symbols denote organic fertilizer regimes, and ellipses indicate descriptive 95% multivariate-t distribution regions within each season. CK, control; SM, sheep manure; PM, pig manure; RM, rapeseed cake manure; OF, commercial organic fertilizer.

Principal coordinate analysis (PCoA) based on Bray–Curtis dissimilarities revealed distinct response patterns of bacterial and fungal communities to organic fertilization regime and seasonal variation (Figure 5). These patterns were subsequently quantified using PERMANOVA with restricted permutations; complete test statistics, including df, SS, R^2^, pseudo-F, and *p*-values, are reported in Table 3. Potential differences in multivariate dispersion were evaluated using the betadisper procedure (Table S4).

The bacterial community exhibited a pronounced seasonal gradient, with spring samples clearly separated from those collected in summer and autumn, whereas samples subjected to different organic fertilization regimes overlapped substantially within each season (Figure 5A). Restricted-permutation PERMANOVA indicated that season was the primary source of variation in bacterial community composition (R^2^ = 0.6870, pseudo-F = 59.713, *p* = 0.0001), while fertilization regime explained a smaller but significant proportion of the variation (R^2^ = 0.0651, pseudo-F = 2.828, *p* = 0.0001; Table 3). The fertilization regime × season interaction was not significant (R^2^ = 0.0753, pseudo-F = 1.637, *p* = 0.2976), indicating that seasonal shifts in bacterial community composition did not differ significantly among fertilization regimes.

The betadisper analysis detected no significant differences in bacterial multivariate dispersion among fertilization regimes or among fertilization regime × season combinations. However, dispersion differed significantly among seasons (Table S4). Thus, the effect of fertilization regime primarily reflected shifts in community centroids. In contrast, the strong seasonal effect should be interpreted with caution because it may reflect both separation among seasonal centroids and differences in within-season dispersion, which cannot be disentangled by the overall PERMANOVA test alone.

Compared with bacterial communities, fungal communities showed differentiation associated with both season and organic fertilization regime in the PCoA ordination (Figure 5B). PERMANOVA further confirmed significant effects of both factors. Season explained 27.33% of the variation in fungal community composition (R^2^ = 0.2733, pseudo-F = 10.716, *p* = 0.0001), whereas fertilization regime explained 18.61% (R^2^ = 0.1861, pseudo-F = 3.649, *p* = 0.0001; Table 3). Therefore, although season remained the stronger predictor, the relative contribution of fertilization regime was substantially greater for fungal communities than for bacterial communities. The fertilization regime × season interaction was not significant (R^2^ = 0.1580, pseudo-F = 1.549, *p* = 0.1234), again indicating that seasonal changes in fungal community composition did not differ significantly among fertilization regimes.

The betadisper analysis showed no significant differences in fungal multivariate dispersion among fertilization regimes, seasons, or fertilization regime × season combinations (Table S4). Accordingly, the significant main effects primarily reflected shifts in the locations of fungal community centroids rather than differences in within-group dispersion.

Overall, the structures of both bacterial and fungal communities were significantly associated with fertilization regime and season, although the relative contributions of these factors differed between the two microbial groups. Bacterial β-diversity was driven predominantly by season, with a weaker but significant effect of fertilization regime, whereas fungal β-diversity showed substantial variation associated with both season and fertilization regime. No significant fertilization regime × season interaction was detected for either microbial group.

### 3.5. Exploratory Screening of Treatment-Associated Genera

**Figure 6:**
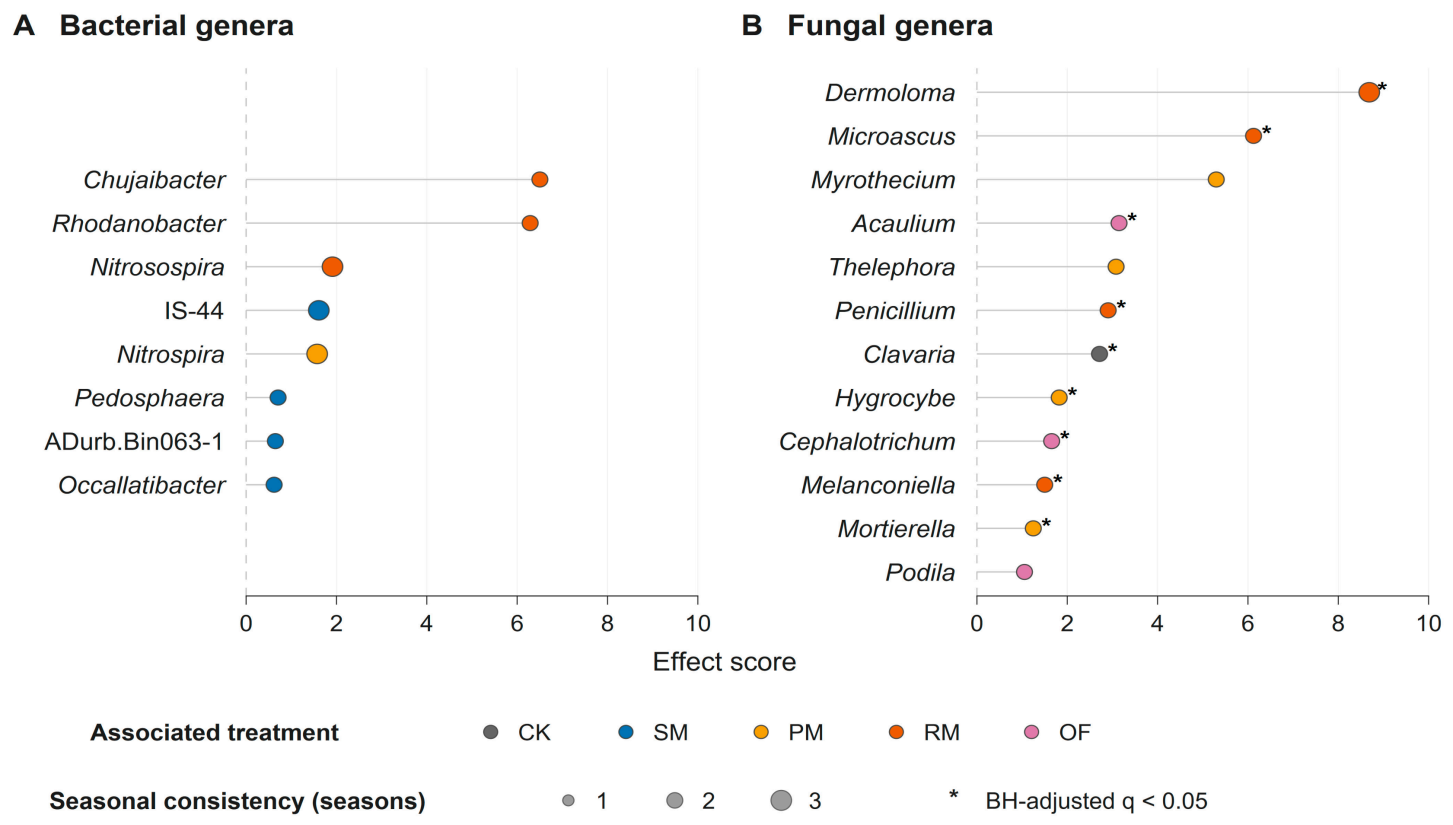
Exploratory genus-level screening across organic fertilizer regimes. (**A**) Eight bacterial genera meeting the initial screening criteria, none of which remained significant after FDR correction; (**B**) 12 fungal genera, of which nine remained significant after FDR correction and three were retained as exploratory observations. The effect score is the log2 ratio of the mean relative abundance in the associated treatment to the mean across the other four treatments. Colors denote the associated treatment; point size, the seasonal consistency (number of seasons); and asterisks, the BH-adjusted q < 0.05. CK, control; SM, sheep manure; PM, pig manure; RM, rapeseed cake manure; OF, commercial organic fertilizer.

Treatment-associated patterns in genus-level relative abundance were examined in an exploratory analysis for bacterial communities based on the 16S rRNA gene dataset, which was renormalized after the removal of archaeal taxa, and for fungal communities based on the ITS dataset (Table 4; Figure 6). Taxa with a Kruskal–Wallis *p*-value < 0.05, a clear treatment-associated response pattern, and consistent association directions in at least two seasons were retained for further descriptive assessment. As the latter two criteria were descriptive, taxa that did not remain significant after FDR correction were treated only as exploratory observations. FDR-adjusted *p*-values (q values) were therefore used to distinguish statistically significant treatment-associated differences (q < 0.05) from exploratory observations (q ≥ 0.05). A total of eight bacterial genera and twelve fungal genera met the initial screening criteria. After FDR correction, nine fungal genera remained significantly different among treatments, whereas none of the bacterial genera reached the adjusted significance threshold.

The eight bacterial genera retained in the exploratory screening were primarily associated with the SM and RM treatments (Table 4; Figure 6A). IS-44, *Pedosphaera*, ADurb.Bin063-1, and *Occallatibacter* were associated with SM, whereas *Chujaibacter*, *Rhodanobacter*, and *Nitrosospira* were associated with RM. *Nitrospira* was the only genus retained under PM, while no bacterial genera met the screening criteria under CK or OF. *Chujaibacter* and *Rhodanobacter* showed the largest bacterial effect scores under RM, with values of 6.503 and 6.288, respectively. *Nitrosospira*, IS-44, and *Nitrospira* showed consistent association directions across all three seasons. Although all eight bacterial genera met the initial screening criteria, none remained significant after false discovery rate correction (q ≥ 0.05). These bacterial patterns were therefore treated as exploratory observations rather than statistically significant differential taxa.

Fungal associations were broader and statistically more robust. Among the 12 treatment-associated genera, four were associated with RM, four with PM, three with OF, and one with CK, whereas none were associated with SM (Table 4; Figure 6B). Nine genera retained a significant overall treatment effect after FDR correction (q < 0.05). *Dermoloma*, *Microascus*, *Penicillium*, and *Melanconiella* showed their highest effect scores under RM; *Hygrocybe* and *Mortierella* under PM; *Acaulium* and *Cephalotrichum* under OF; and *Clavaria* under CK. *Dermoloma* and *Microascus* had the two largest fungal effect scores, at 8.686 and 6.124, respectively. Moreover, *Dermoloma* showed a consistent association direction across all three seasons. *Myrothecium* and *Thelephora*, associated with PM, and *Podila*, associated with OF, met the initial screening criteria but did not remain significant after FDR correction and were therefore retained only as exploratory observations.

Overall, genus-level differentiation was more pronounced for fungi than for bacteria. Fungal associations were concentrated mainly under RM, PM, and OF, with nine of the 12 genera remaining significant after multiple-testing correction. In contrast, the bacterial patterns observed mainly under SM and RM did not remain significant after FDR correction and should therefore be interpreted as exploratory.

### 3.6. Environmental Associations with Bacterial and Fungal Community Variation

**Figure 7:**
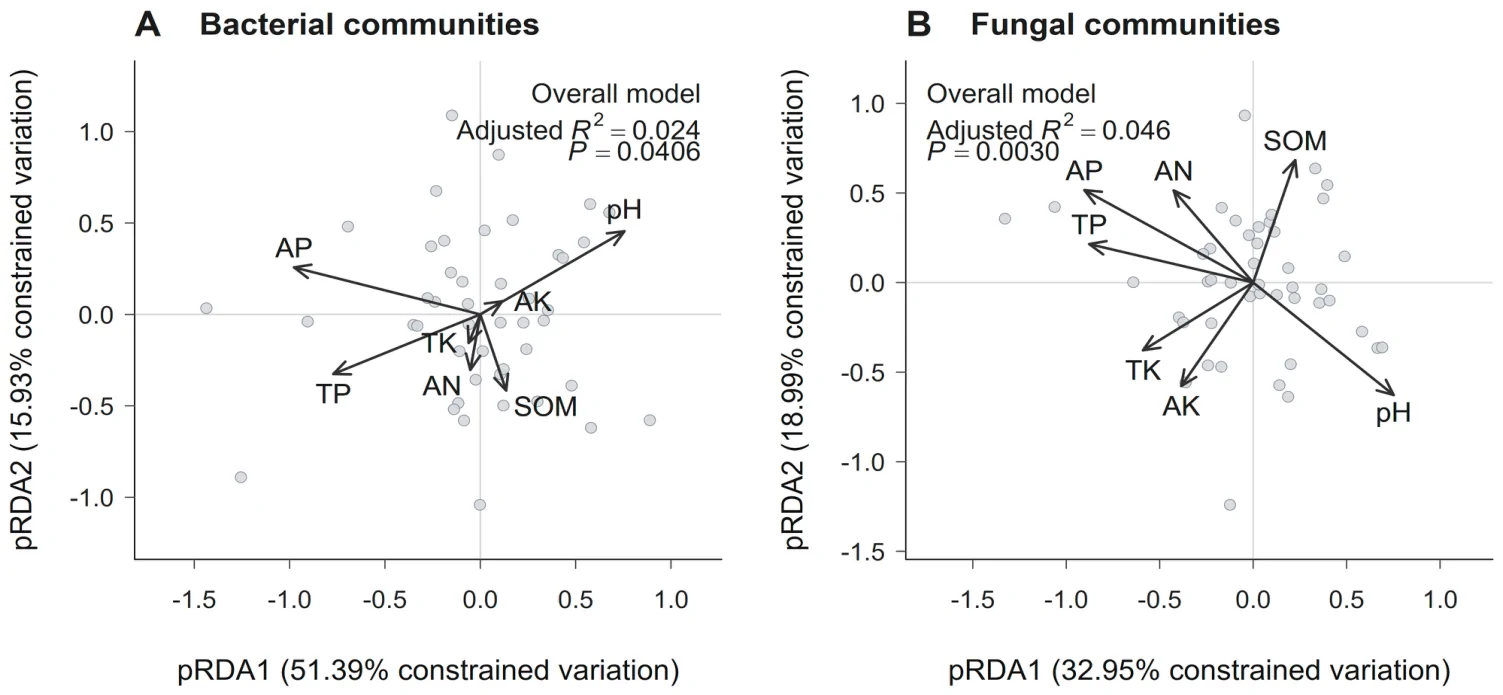
Design-adjusted partial redundancy analysis (pRDA) relating (**A**) bacterial and (**B**) fungal genus-level communities to soil physicochemical properties. Community matrices were Hellinger-transformed, fertilization regime and season were conditioned out, and significance was assessed using 9999 permutations restricted within TreeID. TN was excluded during VIF screening. Gray circles represent individual samples, and arrows show the direction and relative strength of associations for the retained variables (pH, SOM, TP, TK, AN, AP, and AK) within each panel; axis labels give the percentage of constrained variation. Overall-model adjusted R^2^ and *p*-values are shown in each panel. No individual soil variable remained significant after Benjamini–Hochberg FDR correction.

Design-adjusted partial redundancy analysis (pRDA) was used to assess associations between soil physicochemical properties and Hellinger-transformed genus-level community composition after accounting for fertilization regime and season. Permutations (9999) were restricted within TreeID to accommodate repeated sampling, and TN was excluded during variance inflation factor screening because of multicollinearity. The retained edaphic variables (pH, SOM, TP, TK, AN, AP, and AK) jointly explained a small but significant fraction of variation in both bacterial (partial R^2^ = 0.0651, adjusted R^2^ = 0.0241, pseudo-F = 1.470, *p* = 0.0406) and fungal communities (partial R^2^ = 0.1356, adjusted R^2^ = 0.0461, pseudo-F = 1.415, *p* = 0.0030; Figure 7; Table 5). The corresponding explained fraction was therefore larger for fungi than for bacteria.

For bacterial communities, pRDA1 and pRDA2 captured 51.39% and 15.93% of the constrained variation, respectively (Figure 7A). pH (partial R^2^ = 0.0523, pseudo-F = 2.160, raw *p* = 0.0143) and AP (partial R^2^ = 0.0453, pseudo-F = 1.871, raw *p* = 0.0312) showed the strongest nominal associations. Neither term remained significant after FDR correction (FDR-adjusted *p* = 0.1001 and 0.1092, respectively).

For fungal communities, pRDA1 and pRDA2 captured 32.95% and 18.99% of the constrained variation, respectively (Figure 7B). The strongest nominal associations were observed for pH (partial R^2^ = 0.0418, pseudo-F = 1.709, raw *p* = 0.0318) and AN (partial R^2^ = 0.0343, pseudo-F = 1.404, raw *p* = 0.0330), but neither remained significant after FDR correction (FDR-adjusted *p* = 0.1155 for both variables).

Accordingly, the ordinations suggest kingdom-specific tendencies—pH and AP for bacteria and pH and AN for fungi—without identifying a single independently supported edaphic predictor (Table S5). A sensitivity analysis conditioning on TreeID and season yielded the same general pattern: the overall soil-variable models remained significant for bacteria (*p* = 0.0317) and fungi (*p* = 0.0190), whereas no individual variable survived FDR correction. Overall, the pRDA analyses supported a collective association between soil physicochemical conditions and microbial community composition, without identifying an independently significant soil predictor.

### 3.7. Prediction of Potential Functional Characteristics of Microbial Communities

**Figure 8:**
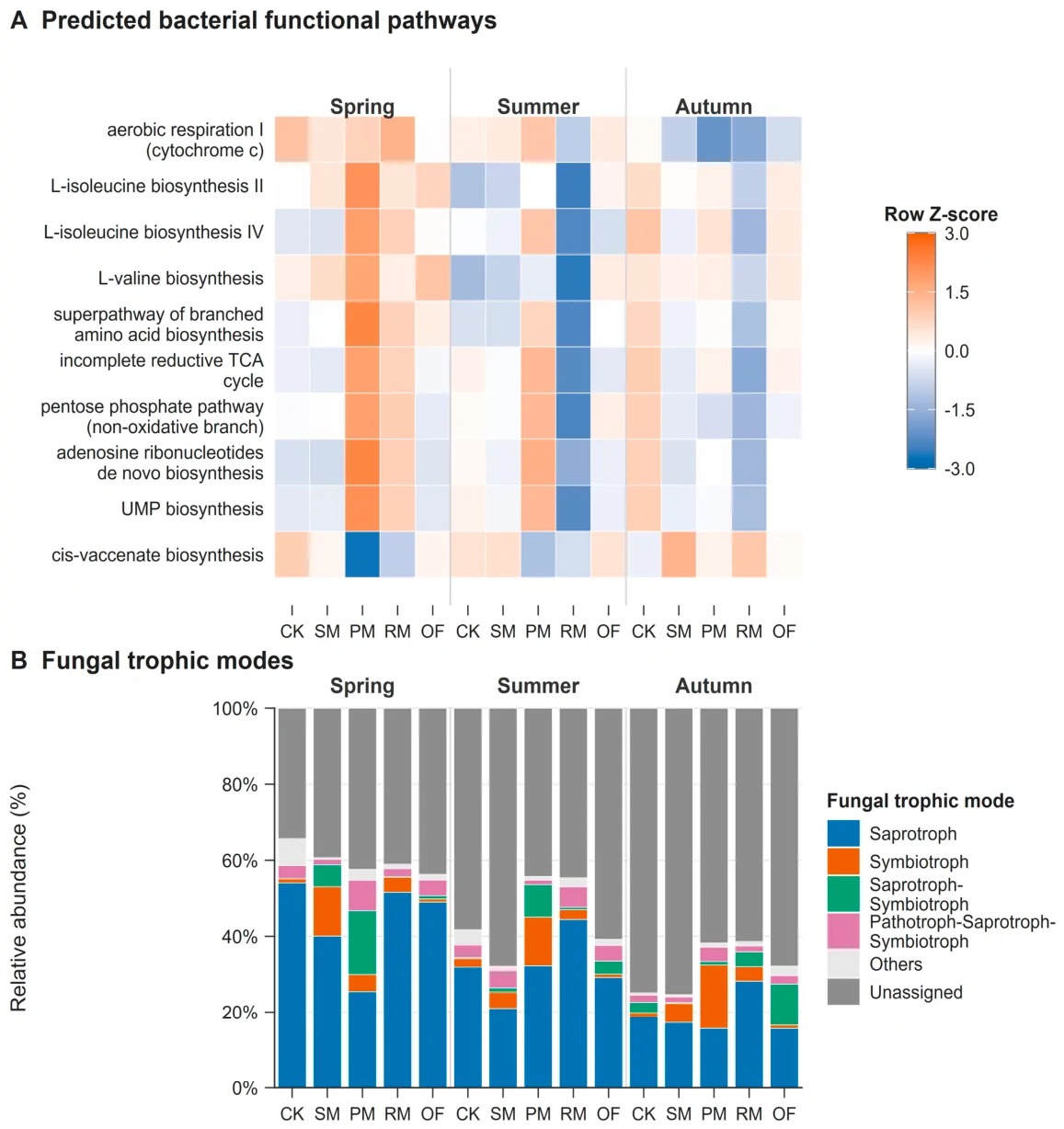
Predicted functional potential of soil bacterial and fungal communities across organic fertilizer regimes and seasons. (**A**) Row-standardized heatmap (Z-scores) of the 10 displayed MetaCyc pathways inferred with PICRUSt2 from the bacteria-only 16S rRNA gene dataset. Colors indicate relative patterns within each pathway and do not represent statistical significance. (**B**) Relative abundances of fungal trophic modes assigned with FUNGuild from the ITS dataset across the 15 combinations of season and fertilization regime. Bars sum to 100%, and “Others” represents low-abundance assigned categories pooled in the plotting dataset. ”Unassigned” denotes taxa for which FUNGuild did not return a trophic-mode assignment and is not itself an ecological guild. CK, control; SM, sheep manure; PM, pig manure; RM, rapeseed cake manure; OF, commercial organic fertilizer.

Bacterial metabolic potential and fungal trophic modes were inferred to characterize putative functional variation among the 15 combinations of season and fertilization regime (Figure 8). PICRUSt2 predictions were generated from the bacteria-only 16S rRNA gene dataset after archaeal ASVs were excluded, whereas fungal trophic modes were assigned from the ITS dataset using FUNGuild. These outputs represent taxonomically inferred functional potential and should not be equated with measured gene abundance, transcription, or metabolic activity.

The 10 displayed bacterial pathways primarily represented branched-chain amino acid and nucleotide biosynthesis, central carbon metabolism, fatty acid biosynthesis, and aerobic respiration (Figure 8A). Spring_PM showed comparatively high row-wise Z scores for L-isoleucine biosynthesis II and IV, L-valine biosynthesis, the superpathway of branched amino acid biosynthesis, the incomplete reductive TCA cycle, the non-oxidative branch of the pentose phosphate pathway, adenosine ribonucleotide de novo biosynthesis, and UMP biosynthesis. In contrast, Summer_RM showed comparatively low scores for most of these pathways. Cis-vaccenate biosynthesis followed a different pattern, with a comparatively low score in Spring_PM and higher scores in Autumn_SM and several other autumn groups. Aerobic respiration I (cytochrome c) also displayed a distinct profile, with generally higher standardized values in spring and lower values in several autumn fertilization groups. Because the heatmap presents row-standardized values and no pathway-level inferential tests are reported here, these contrasts should be interpreted as relative patterns rather than statistically supported treatment, season, or interaction effects.

The FUNGuild output was dominated by the Unassigned fraction (54.54% on average) and the assigned Saprotroph mode (31.61%), whereas Symbiotroph, Saprotroph-Symbiotroph, Pathotroph–Saprotroph–Symbiotroph, and Others contributed smaller proportions (Figure 8B). Descriptively, the mean Saprotroph proportion declined from 44.00% in spring to 31.74% in summer and 19.08% in autumn, while Unassigned increased from 40.18% to 55.17% and 68.26%, respectively. Among the lower-abundance assigned modes, Symbiotroph was relatively more represented in Spring_SM and Autumn_PM, whereas Saprotroph–Symbiotroph was relatively more represented in Spring_PM and Autumn_OF. The seasonal increase in Unassigned may reflect changes in taxonomic composition and database coverage; therefore, it should not be interpreted as a direct ecological shift in fungal function.

Overall, the taxon-based predictions indicated descriptive variation in bacterial MetaCyc pathway profiles and FUNGuild assignments across the 15 combinations of season and fertilization regime. The most apparent bacterial contrasts involved biosynthetic and central metabolic pathways, whereas the fungal profiles showed a declining saprotrophic fraction from spring to autumn alongside a large and seasonally variable Unassigned component. These patterns provide hypotheses about potential seasonal and fertilization-related functional variation but require validation using independent approaches that assess functional genes, transcripts, or activities, such as shotgun metagenomics, metatranscriptomics, targeted functional-gene quantification, or soil enzyme assays.

## 4. Discussion

Practical organic fertilizer regimes and sampling time were associated with nonuniform responses in soil properties and microbial communities. The principal contribution of this study is therefore design-based and context-specific: it combines locally used fertilizer regimes with repeated seasonal observations of the same trees and analysis approaches that account for within-tree dependence. Comparable studies in tea plantations have shown that organic management can alter bacterial and fungal community composition and network stability [43]. Metagenomic evidence from karst tea plantations likewise indicates that management systems can restructure microbial communities and co-occurrence patterns [44]. Long-term organic management has also been linked to soil quality, key taxa, and multifunctionality [45], while equal-N comparisons show changes in soil organic matter, available nutrients, and bacterial communities [46]. Organic management can shift nitrogen transformation [47] and phosphorus solubilization [48]. Six-year field evidence additionally relates prolonged organic fertilizer use to soil properties, bacterial community composition, tea yield, and quality [49]. In the present experiment, AN, AP, and AK showed the clearest treatment-dependent and seasonal variation, but treatments were applied at locally used per-tree rates and were not equalized for N, P, K, or organic carbon. Treatment contrasts thus represent integrated practical regimes—fertilizer material, dose, mineralization pattern, and substrate quality—rather than isolated effects of fertilizer identity or composition. No regime can therefore be considered universally superior on the basis of these data.

Alpha-diversity responses showed that bacteria and fungi reacted at different ecological levels. Bacterial Shannon diversity was associated with fertilization regime and season, whereas bacterial Chao1 richness varied mainly among regimes; neither fungal index showed a significant model-level response. A global meta-analysis similarly found that organic amendments increased bacterial Shannon and Chao1 indices but had no overall effect on fungal diversity indices [50]. A four-year tea-plantation experiment also reported inconsistent bacterial and fungal diversity and network responses to magnesium fertilization [51]. Comparisons among tea-management systems further show that community structure and metabolic profiles can diverge without uniform changes in all diversity indices [52]. The present results are therefore consistent with rapid bacterial responses to labile resources and rhizosphere conditions, whereas fungal responses were expressed more clearly as compositional reorganization than as a net change in richness or Shannon diversity. Nonsignificant fungal alpha diversity should not be interpreted as fungal insensitivity, and alpha diversity alone is insufficient for evaluating management responses.

Community turnover revealed the clearest kingdom-level contrast. Season explained 68.70% of bacterial variation, compared with 6.51% for fertilization regime. This is ecologically plausible because many bacteria have short generation times and respond rapidly to temperature, moisture, labile carbon, nutrient pulses, root exudation, and litter inputs. Seasonal rhizosphere and bulk-soil surveys link resource changes with microbial turnover [53]. Across forests and grasslands, season and edaphic context differently affect communities and enzyme activities [54], and seasonal variation outweighed throughfall reduction in a subtropical plantation [55]. A year-long agricultural study likewise found stronger temporal trends for bacteria than for fungi within fields [56]. In contrast, fungal variation was more evenly associated with season (27.33%) and fertilization regime (18.61%). Fungi can exploit spatially heterogeneous, chemically complex organic materials through hyphal growth and extracellular enzymes; persistent differences in amendment C:N ratio, polymer composition, and nutrient release may therefore leave a stronger regime-specific imprint. After 10 years of organic fertilization, fungal communities were more sensitive than bacterial communities and varied with aggregate-scale C:N redistribution [57]. Deep-banded organic substrate also modified fungal diversity, consistent with hyphal access to localized carbon resources [58]. These are plausible mechanisms, not direct measurements here. Interpretation of the bacterial seasonal effect also requires caution because dispersion differed among seasons, and the three sampling dates represented increasing intervals after a single fertilizer application. Seasonal change therefore cannot be fully separated from post-application succession. The nonsignificant regime-by-season interaction indicates broadly shared temporal trajectories rather than evidence that each regime generated a distinct seasonal course.

Taxonomic profiles indicated reorganization within an established phylum-level framework. Acidobacteriota, Proteobacteria, and Chloroflexi remained dominant after archaeal reads were removed, while Ascomycota and Basidiomycota dominated the fungal profiles. Mountainous tea plantations show comparable dominant groups and lower-rank cultivation-associated shifts [59], and a recent synthesis identifies these lineages as recurrent tea-soil components [60]. Persistence of these phyla therefore does not contradict the significant beta-diversity results; much of the response occurred within dominant lineages or at lower ranks. At genus level, eight bacterial genera met the initial screening criteria, but none remained significant after FDR correction and they should remain exploratory. Nine of 12 treatment-associated fungal genera retained FDR-adjusted significance, supporting stronger fungal differentiation without implying improved function. Each treatment, however, contained only three independent trees. Repeated sampling improved temporal estimation but did not increase independent experimental units. Limited replication reduces power for detecting taxa and increases effect-size uncertainty after FDR correction. Thus, nonsignificant bacterial genera do not prove absence of response, and the fungal taxa require validation with greater field replication.

The design-adjusted pRDA suggests a multivariate environmental context rather than control by a single measured soil property. After conditioning on fertilization regime and season, the retained variables collectively explained 6.51% of bacterial variation (adjusted R^2^ = 0.0241, *p* = 0.0406) and 13.56% of fungal variation (adjusted R^2^ = 0.0461, *p* = 0.0030), but no individual variable remained significant after FDR correction. A multi-year crop-diversification study similarly found that changing combinations of moisture, carbon, nitrogen, microbial biomass, and enzyme indicators jointly explained modest, time-dependent fractions of bacterial and fungal community variation [61]. In the present dataset, pH, SOM, AN, AP, and AK may all contribute to the environmental context, but their independent effects cannot be separated from covariance among soil properties and design factors. Consequently, neither pH nor any single nutrient should be described as the dominant driver. Unmeasured hydrothermal conditions, root activity, substrate chemistry, and mineralization dynamics may also account for residual variation. Because pRDA quantifies conditional associations rather than causation, the effects of individual variables require confirmation through targeted manipulative experiments or long-term studies.

Predicted functional profiles should be interpreted as hypothesis-generating descriptions of taxonomically inferred potential. PICRUSt2 suggested relative variation in biosynthesis, central-carbon, respiration, and fatty-acid-associated pathways, while FUNGuild indicated seasonal changes in trophic-mode composition. Systematic benchmarking has shown that 16S-based metagenome predictions can diverge substantially from matched shotgun metagenomes and that accuracy varies among functions and datasets [62]. The present study did not measure functional genes, transcripts, proteins, metabolites, or enzyme activities, and no pathway-level inferential tests were performed. Moreover, 54.54% of fungal sequences were unassigned on average, limiting the resolution of trophic-mode comparisons. The predictions therefore do not demonstrate altered microbial activity or ecosystem function. Their appropriate use is to identify candidate pathways and guild patterns for subsequent validation by shotgun metagenomics, metatranscriptomics, targeted functional-gene assays, metabolomics, or soil enzyme measurements.

Several limitations define the scope of these conclusions. Fertilizer rates followed local per-tree practice, fertilizer composition was not independently measured, and N, P, K, and organic-carbon inputs were not standardized; observed differences thus combine fertilizer source, dose, and release pattern. Only three independent trees represented each regime, despite repeated sampling, and the experiment covered three dates within one year after a single application without a pretreatment microbial baseline. Temporal patterns therefore integrate season, elapsed time since fertilization, and possible pre-existing differences among trees rather than establishing a stable long-term seasonal cycle. Within these bounds, the study provides a production-relevant comparison and demonstrates the value of jointly analyzing bacterial and fungal responses within a repeated-measures framework. Future studies should compare practical-rate treatments with nutrient-standardized treatments, characterize fertilizer carbon and nutrient composition, increase independent field replication, measure soil temperature and moisture, include pretreatment baselines and multi-year sampling, and directly validate the functional hypotheses.

## 5. Conclusions

Under the locally used, material-specific per-tree application rates tested here, differences among practical fertilization regimes and across sampling seasons were associated with soil nutrient availability and microbial community structure in this mature tea plantation, with the magnitude and direction of responses varying among soil properties and between bacteria and fungi. Alkali-hydrolyzable nitrogen, available phosphorus, and available potassium showed the clearest treatment- and season-associated variation among the measured soil indicators. Bacterial alpha diversity metrics responded more clearly than fungal alpha diversity metrics, and season accounted for 68.70% of bacterial community variation, compared with 6.51% associated with fertilization regime. Fungal community variation was associated more evenly with season (27.33%) and fertilization regime (18.61%). At the genera level, nine fungal genera retained significant treatment-associated differences after FDR correction, whereas the bacterial genus-level patterns remained exploratory. The nonsignificant fertilization regime × season interaction indicates broadly shared temporal trajectories rather than regime-specific seasonal responses.

After accounting for fertilization regime and season, the measured soil properties collectively explained 6.51% of bacterial and 13.56% of fungal community variation, whereas no individual variable remained significant after FDR correction. Accordingly, the data support an association with the combined influence of multiple soil properties and do not identify any single property as a dominant environmental factor. Amplicon-based functional predictions suggested potential variation in bacterial metabolic pathways and fungal trophic modes, but these patterns require direct functional validation. Crucially, application rates followed local practice, fertilizer nutrient composition (N, P, K, and organic carbon) was not independently measured, and nutrient and organic carbon inputs were not standardized; fertilizer type and dose were therefore confounded by design. All treatment contrasts should thus be interpreted as integrated, short-term responses to the practical fertilization regimes tested, not as isolated effects of fertilizer type or composition or as evidence that any fertilizer type is universally superior. The one-year sampling window after a single application also precludes interpreting the temporal patterns as stable long-term seasonality. Within these constraints, season-aware assessment of both bacterial and fungal communities can strengthen context-specific evaluation of practical organic fertilization in tea plantations.

## Figures and Tables

**Table 1:** Effects of organic fertilizer regime, season, and their interaction on soil physicochemical properties based on linear mixed-effects models.

Indicator	Treatment	Season	Treatment × Season
pH	9.609 **	11.340 ***	6.047 ***
SOM	4.626 *	12.220 ***	0.633 ns
TN	10.082 **	10.748 ***	1.406 ns
TP	35.003 ***	15.836 ***	5.631 ***
TK	1.338 ns	1.506 ns	0.829 ns
AN	117.568 ***	0.982 ns	20.453 ***
AP	54.565 ***	8.774 **	5.330 **
AK	7.458 **	35.987 ***	2.650 *

Note: Values are F-values from linear mixed-effects models. * *p* < 0.05; ** *p* < 0.01; *** *p* < 0.001; ns, not significant (*p* ≥ 0.05).

**Table 2:** Effects of organic fertilizer regime, season, and their interaction on alpha diversity indices of bacterial and fungal communities based on linear mixed-effects models.

Microbial Group	Index	Organic Fertilizer Regime	Season	Organic Fertilizer Regime × Season
Bacteria	Shannon	10.537 ** (0.0013)	7.470 ** (0.0038)	2.173 ns (0.0762)
Bacteria	Chao1	10.293 ** (0.0014)	1.724 ns (0.2038)	1.806 ns (0.1354)
Fungi	Shannon	1.150 ns (0.3881)	1.839 ns (0.1849)	0.627 ns (0.7456)
Fungi	Chao1	0.762 ns (0.5732)	1.325 ns (0.2881)	1.376 ns (0.2658)

Note: Values are *F*-values, with corresponding *p*-values in parentheses. ** *p* < 0.01; ns, not significant (*p* ≥ 0.05).

**Table 3:** Restricted-permutation PERMANOVA of the effects of fertilization regime, season, and their interaction on bacterial and fungal community structures. Degrees of freedom (df), sums of squares (SS), R^2^, pseudo-F-values, and *p*-values are reported for each tested term.

Microbial Group	Data Level	Distance	Source	df	SS	R^2^	Pseudo-F	*p*-Value
Bacteria	Genus	Bray–Curtis	Organic fertilizer regime	4	0.3502	0.0651	2.8281	0.0001
Bacteria	Genus	Bray–Curtis	Season	2	3.6969	0.6870	59.7132	0.0001
Bacteria	Genus	Bray–Curtis	Organic fertilizer regime × Season	8	0.4054	0.0753	1.6370	0.2976
Fungi	Genus	Bray–Curtis	Organic fertilizer regime	4	1.0348	0.1861	3.6486	0.0001
Fungi	Genus	Bray–Curtis	Season	2	1.5196	0.2733	10.7157	0.0001
Fungi	Genus	Bray–Curtis	Organic fertilizer regime × Season	8	0.8786	0.1580	1.5489	0.1234

**Table 4:** Results of the exploratory screening of treatment-associated bacterial and fungal genera under different organic fertilizer regimes. Taxa with q < 0.05 were considered to show statistically significant treatment-associated differences, whereas taxa with *p* < 0.05 but q ≥ 0.05 were retained only as exploratory observations.

Microbial Group	Taxon	Associated Treatment	Effect Score	*p*-Value	FDR-Adjusted *p*-Value	Significance (FDR < 0.05)
Bacteria	*Chujaibacter*	RM	6.502996	0.001262	0.053235	ns
Bacteria	*Rhodanobacter*	RM	6.287718	0.005546	0.085964	ns
Bacteria	*Nitrosospira*	RM	1.911827	0.011238	0.116123	ns
Bacteria	*IS-44*	SM	1.608651	0.001942	0.053235	ns
Bacteria	*Nitrospira*	PM	1.572401	0.002576	0.053235	ns
Bacteria	*Pedosphaera*	SM	0.707094	0.026073	0.230929	ns
Bacteria	*ADurb.Bin063-1*	SM	0.647050	0.010400	0.116123	ns
Bacteria	*Occallatibacter*	SM	0.619073	0.047107	0.324514	ns
Fungi	*Dermoloma*	RM	8.685603	0.000001	0.000028	Significant
Fungi	*Microascus*	RM	6.123550	0.000004	0.000068	Significant
Fungi	*Myrothecium*	PM	5.298394	0.020601	0.053880	ns
Fungi	*Acaulium*	OF	3.145603	0.000392	0.003336	Significant
Fungi	*Thelephora*	PM	3.080613	0.048978	0.097957	ns
Fungi	*Penicillium*	RM	2.905579	0.003042	0.014774	Significant
Fungi	*Clavaria*	CK	2.713944	0.000205	0.002322	Significant
Fungi	*Hygrocybe*	PM	1.821317	0.005686	0.021481	Significant
Fungi	*Cephalotrichum*	OF	1.654502	0.000856	0.005820	Significant
Fungi	*Melanconiella*	RM	1.500740	0.001749	0.009912	Significant
Fungi	*Mortierella*	PM	1.247694	0.006705	0.022799	Significant
Fungi	*Podila*	OF	1.053097	0.032931	0.069979	ns

Note: ns, not significant after FDR correction (FDR-adjusted *p* ≥ 0.05).

**Table 5:** Overall significance of design-adjusted partial redundancy analysis for bacterial and fungal communities.

Microbial Group	Conditioning Variables	Partial R^2^	Adjusted R^2^	Pseudo-F	*p*-Value
Bacteria	Treatment + Season	0.0651	0.0241	1.470	0.0406
Fungi	Treatment + Season	0.1356	0.0461	1.415	0.0030

## Data Availability

The raw data supporting the conclusions of this article will be made available by the authors on request. The processed data supporting the findings of this study, including soil physicochemical properties, diversity indices, community composition tables, and statistical results, are available within the article and its Supplementary Materials. Additional data are available from the corresponding author upon reasonable request.

## Supplementary Materials

The following supporting information can be downloaded at: https://www.mdpi.com/article/10.3390/microorganisms14091902/s1, Figure S1: Rarefaction curves of bacterial and fungal communities; Figure S2: ASV accumulation curves of bacterial and fungal communities; Figure S3: Spearman correlations between treatment-associated genera retained in the exploratory screening and soil physicochemical properties; Table S1: Sequencing depth and quality statistics of 16S rRNA gene amplicon data; Table S2: Sequencing depth and quality statistics of fungal ITS amplicon data; Table S3: Fertilizer materials, sources, application rates, and availability of composition data for the five treatments; Table S4: Homogeneity of multivariate dispersion tested by betadisper; Table S5: Marginal effects of individual soil physicochemical variables in design-adjusted partial redundancy analysis of bacterial and fungal communities.

## References

[1] Yan P., Wu L., Wang D., Fu J., Shen C., Li X., Zhang L., Zhang L., Fan L., Huang W. (2020). Soil acidification in Chinese tea plantations. Sci. Total Environ..

[2] Zhao J., Fu J., Liu N., Li X., Zan L., Yan F., Qu D. (2023). Research progress on fertilization in tea plants. Soils Fertil. Sci. China.

[3] Liu B., Zhang Y., Yi X., Zheng H., Ni K., Ma Q., Cai Y., Ma L., Shi Y., Yang X. (2025). Partially replacing chemical fertilizer with manure improves soil quality and ecosystem multifunctionality in a tea plantation. Agric. Ecosyst. Environ..

[4] Wang X., Zhao N., Li W., Pu X., Xu P., Wang P. (2024). Core bacterial taxa determine formation of forage yield in fertilized soil. Microorganisms.

[5] Lei Y., Ding D., Duan J., Luo Y., Huang F., Kang Y., Chen Y., Li S. (2024). Soil microbial community characteristics and their effect on tea quality under different fertilization treatments in two tea plantations. Genes.

[6] Miao P., Pang X., Zhang M., Cheng W., Zhou Z., Li Y., Wang H., Jia X., Ye J., Zhang Q. (2024). Enhancing soil health and tea plant quality through integrated organic and chemical fertilization strategies. Horticulturae.

[7] Jia X., Weng Q., Wang T., Zhang Q., Gu J., Liao Y., Zhu B., Wang H., Ye J. (2025). Fertilizer application drives the restructuring of microbial communities and functional succession in the rhizosphere soil of Camellia sinensis. Horticulturae.

[8] Fu H., Li H., Yin P., Mei H., Li J., Zhou P., Wang Y., Ma Q., Jeyaraj A., Thangaraj K. (2021). Integrated application of rapeseed cake and green manure enhances soil nutrients and microbial communities in tea garden soil. Sustainability.

[9] Zhou P., Chen M., Bao Q., Wang H., Wang Y., Fu H. (2024). The effect of intercropping with different leguminous green manures on the soil environment and tea quality in tea plantations. Microorganisms.

[10] Duan C., Liao H., Su J., Yu C., Mo Y., Wang W., Ren Y. (2026). Microbial inoculant augments the influence of organic fertilizer on soil microbial communities in tea plantations. J. Environ. Manag..

[11] Pu T., Tan Y., Zhao Y., Zhao Z., Zhang N., Li C., Song Y. (2025). Effect of seasonal variations on soil microbial, extracellular enzymes, and ecological stoichiometry in tea plantations. Ecol. Evol..

[12] Yang Y., Kim J., Chung J.O., Cho D., Roh J.H., Hong Y.D., Kim W.G., Kang H. (2022). Variations in the composition of tea leaves and soil microbial community. Biol. Fertil. Soils.

[13] Kui L., Xiang G., Wang Y., Wang Z., Li G., Li D., Yan J., Ye S., Wang C., Yang L. (2021). Large-scale characterization of the soil microbiome in ancient tea plantations using high-throughput 16S rRNA and internal transcribed spacer amplicon sequencing. Front. Microbiol..

[14] Yang X., Huang X., Hu X., Cheng X., Luo Y. (2024). Changes in rhizosphere and bulk soil microbial communities of tableland tea garden and ancient tea plantation in Southwest China. Agronomy.

[15] (2007). Determination of pH in Soil.

[16] (2006). Soil Testing—Part 6: Determination of Soil Organic Matter.

[17] (2012). Soil Testing—Part 24: Determination of Total Nitrogen in Soil—Automatic Kjeldahl Apparatus Method.

[18] (2015). Phosphorus Determination Methods of Forest Soils.

[19] (2015). Potassium Determination Methods of Forest Soils.

[20] (2014). Determination of Soil Alkali-Hydrolyzable Nitrogen.

[21] (2014). Soil Testing—Part 7: Determination of Available Phosphorus in Soil.

[22] (2004). Determination of Soil Available Potassium and Slowly Available Potassium Content.

[23] Bolyen E., Rideout J.R., Dillon M.R., Bokulich N.A., Abnet C.C., Al-Ghalith G.A., Alexander H., Alm E.J., Arumugam M., Asnicar F. (2019). Reproducible, interactive, scalable and extensible microbiome data science using QIIME 2. Nat. Biotechnol..

[24] Callahan B.J., McMurdie P.J., Rosen M.J., Han A.W., Johnson A.J.A., Holmes S.P. (2016). DADA2: High-resolution sample inference from Illumina amplicon data. Nat. Methods.

[25] Quast C., Pruesse E., Yilmaz P., Gerken J., Schweer T., Yarza P., Peplies J., Glöckner F.O. (2013). The SILVA ribosomal RNA gene database project: Improved data processing and web-based tools. Nucleic Acids Res..

[26] Nilsson R.H., Larsson K.H., Taylor A.F.S., Bengtsson-Palme J., Jeppesen T.S., Schigel D., Kennedy P., Picard K., Glöckner F.O., Tedersoo L. (2019). The UNITE database for molecular identification of fungi: Handling dark taxa and parallel taxonomic classifications. Nucleic Acids Res..

[27] Bates D., Mächler M., Bolker B., Walker S. (2015). Fitting Linear Mixed‑Effects Models Using lme4. J. Stat. Softw..

[28] Legendre P., Gallagher E.D. (2001). Ecologically meaningful transformations for ordination of species data. Oecologia.

[29] Anderson M.J. (2001). A new method for non-parametric multivariate analysis of variance. Austral Ecol..

[30] Anderson M.J., ter Braak C.J.F. (2003). Permutation tests for multi-factorial analysis of variance. J. Stat. Comput. Simul..

[31] Anderson M.J. (2006). Distance-based tests for homogeneity of multivariate dispersions. Biometrics.

[32] Benjamini Y., Hochberg Y. (1995). Controlling the false discovery rate: A practical and powerful approach to multiple testing. J. R. Stat. Soc. Ser. B.

[33] Douglas G.M., Maffei V.J., Zaneveld J.R., Yurgel S.N., Brown J.R., Taylor C.M., Huttenhower C., Langille M.G.I. (2020). PICRUSt2 for prediction of metagenome functions. Nat. Biotechnol..

[34] Nguyen N.H., Song Z., Bates S.T., Branco S., Tedersoo L., Menke J., Schilling J.S., Kennedy P.G. (2016). FUNGuild: An open annotation tool for parsing fungal community datasets by ecological guild. Fungal Ecol..

[35] R Core Team (2025). R: A Language and Environment for Statistical Computing.

[36] Posit Team (2026). RStudio: Integrated Development Environment for R.

[37] Pinheiro J., Bates D., R Core Team (2025). R Package Version 3.1-168; nlme: Linear and Nonlinear Mixed Effects Models.

[38] Kuznetsova A., Brockhoff P.B., Christensen R.H.B. (2017). lmerTest package: Tests in linear mixed effects models. J. Stat. Softw..

[39] Oksanen J., Simpson G.L., Blanchet F.G., Kindt R., Legendre P., Minchin P.R., O’Hara R.B., Solymos P., Stevens M.H.H., Szoecs E. (2025). R Package, Version 2.7-2; vegan: Community Ecology Package.

[40] Simpson G.L. (2025). R Package, Version 0.9-8; permute: Functions for Generating Restricted Permutations of Data.

[41] Wickham H. (2016). ggplot2: Elegant Graphics for Data Analysis.

[42] Kolde R. (2025). R Package, Version 1.0.13; pheatmap: Pretty Heatmaps.

[43] Huang X., Zheng Y., Li P., Cui J., Sui P., Chen Y., Gao W. (2023). Organic management increases beneficial microorganisms and promotes the stability of microecological networks in tea plantation soil. Front. Microbiol..

[44] Pu T., Zhang N., Wang J., Zhao Z., Tan W., Li C., Song Y. (2024). Organic management pattern improves microbial community diversity and alters microbial network structure in karst tea plantation. Heliyon.

[45] Yu T., Wen Y., Zhang Q., Yang J., Zang H., Zeng Z., Yang Y. (2025). Organic management improves soil multifunctionality by enhancing soil quality and enriching key microbes in tea plantation. Appl. Soil Ecol..

[46] Yang J., Chen Z., Dai J., Liu F., Zhu J. (2025). Effects of long-term organic fertilizer application on tea plantation soil of its physical and chemical properties and microbial communities. Pol. J. Environ. Stud..

[47] Huang X., Wang B., Li P., Chen A., Cui J., Chen Y., Gao W. (2025). Organic management promotes nitrogen transformation in tea plantations soil: A case study from southwestern China. Appl. Soil Ecol..

[48] Chang Y., Wu Z., Peñuelas J., Sardans J., Chen Y., Jiang F., Wang F. (2025). Organic management improves soil P availability via increasing inorganic P solubilization in tea plantations. Environ. Technol. Innov..

[49] Dai C., Xiang F., Liu H., Zhou L., Li W. (2025). The prolonged application of organic fertilizers increases the quality and yield of tea crops. Plants.

[50] Cui J., Yang B., Zhang M., Song D., Xu X., Ai C., Liang G., Zhou W. (2023). Investigating the effects of organic amendments on soil microbial composition and its linkage to soil organic carbon: A global meta-analysis. Sci. Total Environ..

[51] Yang W., Ji Z., Wu A., He D., Rensing C., Chen Y., Chen C., Wu H., Muneer M.A., Wu L. (2023). Inconsistent responses of soil bacterial and fungal community diversity and network to magnesium fertilization in tea (*Camellia sinensis*) plantation soils. Appl. Soil Ecol..

[52] Li G., Zhu S., Long J., Mao H., Dong Y., Hou Y. (2023). Differences in microbial community structure and metabolic activity among tea plantation soils under different management strategies. Front. Microbiol..

[53] Xu T., Shen Y., Ding Z., Zhu B. (2023). Seasonal dynamics of microbial communities in rhizosphere and bulk soils of two temperate forests. Rhizosphere.

[54] Li Y., Ma J., Li Y., Shen X., Xia X. (2024). Microbial community and enzyme activity respond differently to seasonal and edaphic factors in forest and grassland ecosystems. Appl. Soil Ecol..

[55] Lin Y., Yang L., Chen Z., Gao Y., Kong J., He Q., Su Y., Li J., Qiu Q. (2023). Seasonal variations of soil bacterial and fungal communities in a subtropical Eucalyptus plantation and their responses to throughfall reduction. Front. Microbiol..

[56] Joos L., Ommeslag S., Baeyen S., Asselberg W., Van Loo K., Clement L., Debode J., Vandecasteele B., De Tender C. (2025). Year-long, multiple-timepoint field studies show the importance of spatiotemporal dynamics and microbial functions in agricultural soil microbiomes. mSystems.

[57] Cui H., Zhu H., Shutes B., Rousseau A.N., Feng W.-D., Hou S.-N., Ou Y., Yan B.-X. (2023). Soil aggregate-driven changes in nutrient redistribution and microbial communities after 10-year organic fertilization. J. Environ. Manag..

[58] Vido J.J., Jin J., Hayden H.L., Celestina C., Sale P.W.G., Armstrong R., Tang C., Wood J.L., Franks A.E. (2024). Modified fungal diversity in dense clay subsoils after deep-banding organic substrate. Soil Res..

[59] Shao S., Li Y., Li Z., Ma X., Zhu Y., Luo Y., Cai P., Jia X., Rensing C., Li Q. (2024). Impact of tea tree cultivation on soil microbiota, soil organic matter, and nitrogen cycling in mountainous plantations. Agronomy.

[60] Jibola-Shittu M.Y., Heng Z., Keyhani N.O., Dang Y., Chen R., Liu S., Lin Y., Lai P., Chen J., Yang C. (2024). Understanding and exploring the diversity of soil microorganisms in tea (*Camellia sinensis*) gardens: Toward sustainable tea production. Front. Microbiol..

[61] Wooliver R., Kivlin S.N., Jagadamma S. (2025). Microbial communities and their association with soil health indicators under row cash crop and cover crop diversification: A case study. Front. Microbiol..

[62] Matchado M.S., Rühlemann M., Reitmeier S., Kacprowski T., Frost F., Haller D., Baumbach J., List M. (2024). On the limits of 16S rRNA gene-based metagenome prediction and functional profiling. Microb. Genom..

